# *Listeria monocytogenes* Cerebritis and Infective Endocarditis in an Immunocompetent Adult: A Rare Clinical Manifestation

**DOI:** 10.1155/2023/7405556

**Published:** 2023-05-22

**Authors:** Shalini A. Mohan, Zharif Sufyaan

**Affiliations:** Department of Internal Medicine, Ampang Hospital, Ampang, Selangor, Malaysia

## Abstract

Cerebritis and infective endocarditis caused by *Listeria monocytogenes* are very rare. A 56-year-old man presented with slurring of speech and generalized body weakness of 1 week duration. He did not have any past medical history. On systemic examination, he had mild slurring of speech and facial asymmetry and was initially treated for multifocal chronic cerebral infarcts. *Listeria monocytogenes* was isolated from blood culture on day 5 of admission. A diagnosis of neurolisteriosis was made as contrast-enhanced-computed tomography (CECT) of the brain showed right frontal cerebritis. He was treated with intravenous benzyl penicillin. His general condition was improving until day 13 of hospitalization whereby he developed haemoptysis and severe Type 1 respiratory failure requiring reintubation. An urgent transthoracic echocardiogram revealed a large vegetation at the anterior mitral valve leaflet measuring 2.01 cm. No active arterial bleeding was seen on computed tomography angiography (CTA) of the thorax. Magnetic resonance imaging (MRI) of the brain showed evidence of right frontal cerebritis. He continued to deteriorate and succumbed to his illness after 3 weeks of hospitalization. Clinicians should be aware of such an occurrence and prompt recognition and adequate treatment are necessary in cases of *Listeria monocytogenes* cerebritis and infective endocarditis as both are deadly entities.

## 1. Introduction

*Listeria monocytogenes* is a Gram-positive bacillus mainly affecting immunocompromised hosts. Meningitis is clinically the most common manifestation of neurolisteriosis. Other less common manifestations include brain abscess, brainstem encephalitis, and cerebritis [1]. Haykal et al. [2], Salata et al. [3], and Milosevic et al. [4] have previously reported cases of *Listeria monocytogenes* cerebritis.

The case fatality rate of neurolisteriosis is about 36%, and when associated with *Listeria monocytogenes* bacteraemia, the mortality rate was significantly increased [5].

Clinicians should be aware that *Listeria monocytogenes* is resistant to third generation cephalosporins, often used empirically to treat central nervous system infections and penicillin group of antibiotics such as benzyl penicillin should be initiated as soon as *L. monocytogenes* is isolated from the cerebrospinal fluid (CSF) or blood culture and is often combined with gentamicin due to the synergistic effect.

On the other hand, infective endocarditis is another rare complication of *L. monocytogenes* bacteraemia occurring both in native and prosthetic valves [6, 7]. Due to the scarcity of these reports, the optimal treatment and outcome of *Listeria* endocarditis have not been completely established.

In this case report, we present a rare case of a patient with *L. monocytogenes* bacteraemia causing neurolisteriosis and infective endocarditis.

## 2. Case Report

A 56-year-old man presented to our emergency room with slurring of speech and generalized body weakness of one week duration. He had no reported medical illness with no history of alcoholism or other immunosuppressive illnesses. He denied history of chest pain, cough, abdominal pain, trauma, ear pain or discharge, toothache, or consumption of raw food.

On physical examination, he remained afebrile and did not exhibit signs of meningeal irritation. The blood gas analysis on room air revealed a pH of 7.5, pco_2_ 28 mmHg, po_2_ 72 mmHg, spo_2_ of 85%, and bicarbonate level of 21.8 mmol/l. Pulmonary, cardiovascular, and abdominal examinations were unremarkable. On neurological exam, he had mild slurring of speech and left facial asymmetry.

Biochemical results were suggestive of infection evidenced by white cell count of 18,600/*μ*L (4.078–11.37) and C-reactive protein of 40 mg/L. Electrocardiograph (ECG) showed no acute ischemic changes; however this patient's cardiac enzyme, troponin I was raised at 1072 ng/L. Chest radiography showed bilateral lower zone consolidations. A computed tomography (CT) of the brain revealed multifocal chronic infarcts.

In our emergency room, he developed worsening respiratory distress, was intubated for impending respiratory collapse, and was treated for non-ST elevation myocardial infarction and multifocal old cerebral infarcts with dual antiplatelet therapy and anticoagulant. This patient was prescribed intravenous ceftriaxone for pneumonia.

He was extubated after 4 days. On day 5 of admission, the blood culture yielded *Listeria monocytogenes* which raised the clinical suspicion of neurolisteriosis. We proceeded with lumbar puncture, and the obtained CSF was clear, where CSF glucose was 5.9 mmol/L (blood glucose 8.0 mmol/L) and CSF protein 0.58 g/L. Listeria PCR for CSF was negative. Contrast-enhanced-computed Tomography (CECT) of the brain revealed right frontal cerebritis. A diagnosis of neurolisteriosis was made and antibiotic therapy was switched to intravenous benzyl penicillin 4 million units (MU) every 4 hourly. IV gentamicin (5 mg/kg/day) was also added as per our national antimicrobial guideline.

Our patient showed marked improvement until day 13 of hospitalization whereby he developed sudden onset haemoptysis, was tachypnoeic, and had to be reintubated. His haemoglobin level remained static at 9.5 g/dL and dual antiplatelets were withheld. An urgent CT pulmonary angiography (CTPA) showed bilateral ground glass opacities with crazy paving pattern and bilateral pleural effusion. There was no CT evidence of pulmonary embolism.

Urgent transthoracic echocardiogram revealed a large vegetation over the anterior mitral valve leaflet measuring 2.01 cm with an ejection fraction of 66%, good left ventricular function, mild aortic regurgitation, and tricuspid regurgitation. Our patient continued to deteriorate further requiring vasopressor support. Cardiothoracic surgery was consulted, and a conservative management was opted in view of the patient's hemodynamic instability.

Computed tomography angiography (CTA) of the Thorax performed six days after clinical deterioration showed improving diffuse ground glass opacities. There was no arterial blush to suggest an active arterial haemorrhage.

**Figure 1:**
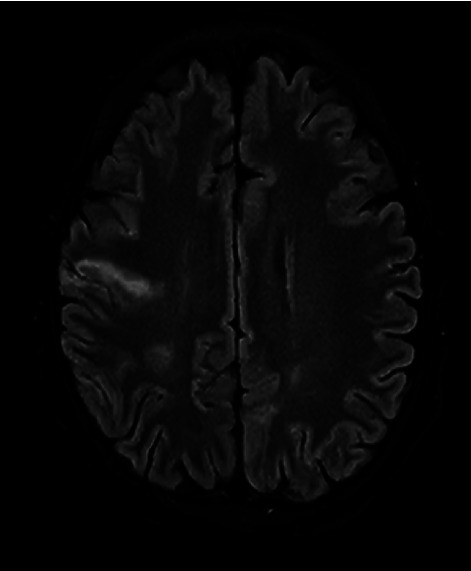
Magnetic resonance imaging (MRI) of the brain on the other hand showed focal gyriform enhancement along the right central sulcus (Rolando's sulcus) with adjacent parenchymal oedema suggestive of cerebritis.

Magnetic resonance imaging (MRI) of the brain on the other hand showed focal gyriform enhancement along the right central sulcus (Rolando's sulcus) with adjacent parenchymal oedema representing cerebritis, associated with adjacent microhaemorrhages. (Figure 1). Our patient unfortunately continued to deteriorate, only to succumb to his illness after 3 weeks of hospitalization.

## 3. Discussion

Charlier et al. reported that listeriosis often presents as an opportunistic infection and is associated with immunosuppressive comorbidities, namely, haematological malignancies or cirrhosis [5]. Our patient, however, was an immunocompetent host and only presented with stroke-like symptoms of one week duration. To our knowledge, there is only another literature by Milosevic et al. in 2018 reporting a case of neurolisteriosis in a 64-year-old immunocompetent host who presented with 16 days history of fever, headache, and middle ear infection, which was not present in our patient [4].

Antal et al. proposed that intraaxonal *L. Monocytogenes* invades the cerebrum and cranial nerves V, VII, IX, X, and XII, gaining entrance from the oropharynx. The bacteria are capable of intraaxonal migration and invade cells, including capillaries of the central nervous system, favouring its spread to the rest of the body [8]. Bacteraemia was an important accompanying feature of *Listeria* cerebritis as reported by Dee and Lorber who reviewed eight cases of multiple cerebral abscesses. In all cases, the etiologic agent was isolated from blood culture, like in the present case report [9].

Similarly, in the study by Charlier et al. in 2017 which included 427 patients with bacteraemia, 87% had a fever and 97% of those with neurolisteriosis were febrile in contrary to our patient. Blood cultures were positive in 63% of those with neurolisteriosis. CSF culture was positive in 84% of those with neurologic presentation. PCR-CSF testing was positive in 63% of those with neurologic presentation (only 16 cases had PCR testing) [5].

The most probable mechanism of development of cerebritis in the present case would be the fact that *Listeria* typically enters the body through the gastrointestinal tract, after ingestion of contaminated food such as cheese, dairy products, and processed food. The bacterium crosses M-cells of Peyer patches and invades the mesenteric lymph nodes, later gaining access to the bloodstream via the reticuloendothelial system. Hepatocytes can promote monocyte recruitment via Toll-like receptor 2 (TLR2)-dependent secretion of CCL2 and CXCL1 chemokines, resulting in the formation of microabscesses [10].

Watson et al. proposed high-dose penicillin or ampicillin therapy for four to six weeks for *Listeria* cerebritis [11]. The combination of ampicillin with gentamicin is generally recommended as a first-line therapy for the treatment of listeriosis. In cases of penicillin hypersensitivity, cotrimoxazole is the treatment of choice.

On the other hand, listerial endocarditis was first described by Hoeprich and Chernoff in 1955 and has since been a changing disease. Most patients with listerial endocarditis did not have a history of exposure to contaminated food or materials. As reported by Fernández Guerrero et al., listerial endocarditis also predominantly affected the immunocompromised hosts. From a total of 68 patients, 28 patients (41.1%) in this review had chronic underlying diseases such as diabetes mellitus, alcoholism, or liver cirrhosis, and at least 11 patients (16.2%) were severely immunocompromised by solid organ transplantation, leukaemia or lymphoma, corticosteroid therapy, haemodialysis, or AIDS, in contrary to our immunocompetent patient [7].

Listerial endocarditis most often occurred on abnormal native or prosthetic valves, mainly on the aortic, mitral, or both valves. The pathological findings were those of a vegetation, with dehiscence of the prosthesis or myocardial abscess formation, fistulisation, and pericarditis [12].

The combined medical and surgical approach seemed to reduce the mortality of patients with endocarditis due to *L. monocytogenes.* Fernández Guerrero et al. reported that of the 59 patients in this review where treatment was clearly stated, 41 were treated with antibiotics alone and 18 received antibiotics and a valve replacement. Sixteen (39.0%) and four (22.2%) patients, respectively, died. In patients with prosthetic valve endocarditis, 63.6% were cured with antibiotics alone and 90.9% were cured with antibiotics plus valve replacement (*p*=0.12). In this review, 23 of 35 (65.7%) patients treated with the combination of penicillin or ampicillin with aminoglycosides survived the infection in comparison with ten out of 14 (71.4%) treated with penicillin monotherapy, exhibiting noninferiority of penicillin monotherapy versus penicillin and aminoglycoside synergism [7]. Our patient unfortunately could not benefit from any surgical approach in view of his hemodynamic instability.

## 4. Conclusion

This case report, while being uncommon, serves as evidence that *Listeria* cerebritis and infective endocarditis may occur in immunocompetent patients. High clinical suspicion, early recognition, and adequate treatment play a pivotal role in improving the outcomes of patients with listeriosis. There is scarce data in the literature and further research in this area, and particularly, the pathogenesis of listeriosis in immunocompetent patients is required.

## Data Availability

The data used to support the findings of this study are available from the corresponding author upon request.
